# Supplementary material for: Did a bot eat your homework? An assessment of the potential impact of bad actors in online administration of preference surveys
Source: PLoS One. 2023 Oct 5;18(10):e0287766. doi: 10.1371/journal.pone.0287766 (PMC10553355; doi:10.1371/journal.pone.0287766)
Supplement: S1 Appendix — (DOCX) [file pone.0287766.s001.docx]

# Appendix A. Results from preference models

**Table A2. Latent Class results**

| **Class 1** | | **Mean** | **SE** | **Z-Score** |
| --- | --- | --- | --- | --- |
| **Route** | **Oral** | 0.006 | 0.046 | 0.130 |
|  | **Injection** | 0.086 | 0.047 | 1.846 |
|  | **IV** | -0.092 | 0.082 | -1.120 |
|  |  |  |  |  |
| **Type** | **CAR-T** | 0.074 | 0.069 | 1.070 |
|  | **BiTE** | 0.013 | 0.066 | 0.197 |
|  | **ADC** | -0.087 | 0.117 | -0.745 |
|  |  |  |  |  |
| **Complete response** | **50%** | 0.350 | 0.044 | 8.022 |
|  | **20%** | -0.039 | 0.044 | -0.900 |
|  | **None** | -0.311 | 0.093 | -3.348 |
|  |  |  |  |  |
| **PFS** | **30 months** | 0.039 | 0.052 | 0.751 |
|  | **18 months** | 0.042 | 0.054 | 0.778 |
|  | **12 months** | -0.081 | 0.092 | -0.880 |
|  |  |  |  |  |
| **Insomnia** | **No insomnia** | -0.060 | 0.048 | -1.237 |
|  | **Mild insomnia** | 0.056 | 0.049 | 1.147 |
|  | **Severe insomnia** | 0.004 | 0.086 | 0.050 |
|  |  |  |  |  |
| **GI Problems** | **No stomach problems** | 0.102 | 0.049 | 2.097 |
|  | **Severe diarrhea** | -0.056 | 0.046 | -1.207 |
|  | **Severe nausea** | -0.046 | 0.081 | -0.574 |
|  |  |  |  |  |
| **Neurotoxicity** | **No neurotoxicity** | 0.140 | 0.047 | 2.966 |
|  | **Neuropathy** | 0.025 | 0.049 | 0.503 |
|  | **CNS neurotoxicity** | -0.165 | 0.085 | -1.932 |
|  |  |  |  |  |
| **Risk of CRS** | **No risk** | 0.130 | 0.045 | 2.918 |
|  | **3%** | 0.009 | 0.044 | 0.196 |
|  | **5%** | -0.056 | 0.063 | -0.899 |
|  | **10%** | -0.083 | 0.087 | -0.945 |
|  |  |  |  |  |
| **Class 2** | | **Mean** | **SE** | **Z-Score** |
| **Route** | **Oral** | -0.311 | 0.411 | -0.757 |
|  | **Injection** | 0.181 | 0.400 | 0.451 |
|  | **IV** | 0.131 | 0.261 | 0.500 |
|  |  |  |  |  |
| **Type** | **CAR-T** | 0.098 | 0.264 | 0.372 |
|  | **BiTE** | -0.119 | 0.232 | -0.512 |
|  | **ADC** | 0.020 | 0.259 | 0.079 |
|  |  |  |  |  |
| **Complete response** | **50%** | -0.093 | 0.174 | -0.534 |
|  | **20%** | 0.434 | 0.229 | 1.892 |
|  | **None** | -0.341 | 0.304 | -1.120 |
|  |  |  |  |  |
| **PFS** | **30 months** | -0.124 | 0.332 | -0.372 |
|  | **18 months** | 0.287 | 0.271 | 1.058 |
|  | **12 months** | -0.163 | 0.284 | -0.575 |
|  |  |  |  |  |
| **Insomnia** | **No insomnia** | 0.027 | 0.383 | 0.071 |
|  | **Mild insomnia** | -0.494 | 0.376 | -1.314 |
|  | **Severe insomnia** | 0.467 | 0.300 | 1.554 |
|  |  |  |  |  |
| **GI Problems** | **No stomach problems** | 0.139 | 0.259 | 0.537 |
|  | **Severe diarrhea** | 0.200 | 0.242 | 0.826 |
|  | **Severe nausea** | -0.339 | 0.297 | -1.141 |
|  |  |  |  |  |
| **Neurotoxicity** | **No neurotoxicity** | -0.644 | 0.445 | -1.448 |
|  | **Neuropathy** | 0.603 | 0.333 | 1.807 |
|  | **CNS neurotoxicity** | 0.041 | 0.285 | 0.143 |
|  |  |  |  |  |
| **Risk of CRS** | **No risk** | 0.288 | 0.205 | 1.408 |
|  | **3%** | 0.015 | 0.240 | 0.061 |
|  | **5%** | -0.228 | 0.234 | -0.975 |
|  | **10%** | -0.074 | 0.297 | -0.251 |
| **Class 1 Membership** | | **Mean** | **SE** | **Z-Score** |
| **scrcount** | | -0.122 | 0.045 | -2.720 |
| **Compcount** | | -0.330 | 0.096 | -3.420 |
| **Time** | | -0.114 | 0.655 | -0.170 |
| **Age** | | 0.021 | 0.017 | 1.240 |
| **stem** | | 0.171 | 0.312 | 0.550 |
| **switch** | | 0.256 | 0.426 | 0.600 |
| **Constant** | | 1.831 | 0.919 | 1.990 |

IV = Intravenous; CAR-T = Chimeric Antigen Receptor; BiTE = Bispecific T-Cell Engager; ADC = Anti-Drug Conjugate PFS = Progression-free survival; GI = Gastrointestinal, CNS = Central Nervous System; CRS = Cytokine Release Syndrome.

**Table A2. RPL results**

| **Mean preferences** | | **Estimate** | **SE** | **Z-score** |
| --- | --- | --- | --- | --- |
| **Route** | **Oral** | 0.036 | 0.065 | 0.549 |
|  | **Injection** | 0.105 | 0.063 | 1.652 |
|  | **IV** | -0.141 | 0.069 | -2.048 |
|  |  |  |  |  |
| **Type** | **CAR-T** | 0.111 | 0.090 | 1.240 |
|  | **BiTE** | -0.066 | 0.097 | -0.675 |
|  | **ADC** | -0.045 | 0.095 | -0.479 |
|  |  |  |  |  |
| **Complete response** | **50%** | 0.563 | 0.095 | 5.934 |
|  | **20%** | -0.050 | 0.066 | -0.755 |
|  | **None** | -0.514 | 0.114 | -4.493 |
|  |  |  |  |  |
| **PFS** | **30 months** | 0.072 | 0.071 | 1.010 |
|  | **18 months** | 0.057 | 0.068 | 0.839 |
|  | **12 months** | -0.129 | 0.068 | -1.903 |
|  |  |  |  |  |
| **Insomnia** | **No insomnia** | -0.066 | 0.065 | -1.021 |
|  | **Mild insomnia** | 0.081 | 0.071 | 1.143 |
|  | **Severe insomnia** | -0.015 | 0.063 | -0.233 |
|  |  |  |  |  |
| **GI Problems** | **No stomach problems** | 0.132 | 0.066 | 2.019 |
|  | **Severe diarrhea** | -0.088 | 0.064 | -1.366 |
|  | **Severe nausea** | -0.045 | 0.064 | -0.692 |
|  |  |  |  |  |
| **Neurotoxicity** | **No neurotoxicity** | 0.209 | 0.065 | 3.189 |
|  | **Neuropathy** | 0.021 | 0.064 | 0.326 |
|  | **CNS neurotoxicity** | -0.229 | 0.073 | -3.160 |
|  |  |  |  |  |
| **Risk of CRS** | **No risk** | 0.156 | 0.060 | 2.604 |
|  | **3%** | 0.048 | 0.062 | 0.782 |
|  | **5%** | -0.091 | 0.090 | -1.011 |
|  | **10%** | -0.114 | 0.092 | -1.241 |
| **Standard deviations** | | **Estimate** | **Estimate** | **SE** |
| **Route** | **Oral** | 0.182 | 0.189 | 0.960 |
|  | **Injection** | -0.232 | 0.166 | -1.400 |
|  | **IV** | 0.051 | 0.265 | 0.190 |
|  |  |  |  |  |
| **Type** | **CAR-T** | 0.271 | 0.278 | 0.980 |
|  | **BiTE** | 0.611 | 0.173 | 3.520 |
|  | **ADC** | -0.882 | 0.348 | -2.540 |
|  |  |  |  |  |
| **Complete response** | **50%** | 0.941 | 0.115 | 8.170 |
|  | **20%** | 0.440 | 0.112 | 3.930 |
|  | **None** | -1.381 | 0.183 | -7.540 |
|  |  |  |  |  |
| **PFS** | **30 months** | 0.283 | 0.145 | 1.960 |
|  | **18 months** | 0.078 | 0.130 | 0.600 |
|  | **12 months** | -0.361 | 0.191 | -1.890 |
|  |  |  |  |  |
| **Insomnia** | **No insomnia** | 0.089 | 0.224 | 0.400 |
|  | **Mild insomnia** | 0.316 | 0.119 | 2.650 |
|  | **Severe insomnia** | -0.405 | 0.246 | -1.640 |
|  |  |  |  |  |
| **GI Problems** | **No stomach problems** | 0.131 | 0.142 | 0.930 |
|  | **Severe diarrhea** | -0.004 | 0.122 | -0.030 |
|  | **Severe nausea** | -0.127 | 0.162 | -0.790 |
|  |  |  |  |  |
| **Neurotoxicity** | **No neurotoxicity** | -0.247 | 0.131 | -1.890 |
|  | **Neuropathy** | 0.170 | 0.117 | 1.450 |
|  | **CNS neurotoxicity** | 0.078 | 0.157 | 0.500 |
|  |  |  |  |  |
| **Risk of CRS** | **No risk** | 0.045 | 0.369 | 0.120 |
|  | **3%** | 0.067 | 0.107 | 0.620 |
|  | **5%** | -0.413 | 0.150 | -2.740 |
|  | **10%** | 0.301 | 0.458 | 0.660 |

IV = Intravenous; CAR-T = Chimeric Antigen Receptor; BiTE = Bispecific T-Cell Engager; ADC = Anti-Drug Conjugate PFS = Progression-free survival; GI = Gastrointestinal, CNS = Central Nervous System; CRS = Cytokine Release Syndrome.
